# Golf as a Physical Activity to Potentially Reduce the Risk of Falls in Older Adults with Parkinson’s Disease

**DOI:** 10.3390/sports9060072

**Published:** 2021-05-23

**Authors:** Rebecca R. Bliss, Frank C. Church

**Affiliations:** 1 Fyzical Therapy and Balance Centers, Durham, NC 27704, USA; rbliss.dpt@gmail.com; 2 Department of Pathology and Laboratory Medicine, The University of North Carolina at Chapel Hill School of Medicine, Chapel Hill, NC 27599, USA

**Keywords:** Parkinson’s disease, golf, older adults, risk of falls, axial mobility, postural instability, bradykinesia, exercise

## Abstract

Advanced age is associated with an increased risk for falls in aging adults. Older adults are also more likely to be diagnosed with Parkinson’s disease (PD), with advanced age as the most significant risk factor. PD is a neurodegenerative disorder with four Cardinal motor symptoms: rigidity, bradykinesia, postural instability, and tremor. Thus, people (person)-with-Parkinson’s disease (PwP) have an even greater risk of falling than non-disorder age-matched peers. Exercise is an activity requiring physical effort, typically carried out to sustain or improve overall health and fitness, and it lowers the risk of falls in the general population. The sport of golf provides a low-impact all-around workout promoting a range of motion, activation of muscles in the upper and lower body, flexibility, and balance. Swinging a golf club offers a unique combination of high amplitude axial rotation, strengthening postural musculature, coordination, and stabilization, demonstrating the potential to impact PD symptoms positively. Golf may be a novel exercise treatment regimen for PD to use in conjunction with traditional medical therapy. We completed a literature review to determine the relationship between the game of golf, PD, and the risk of falls. We concluded that regularly playing golf can lower the risk for falls in community ambulating older adults with PD and demonstrates the potential to improve quality of life for PwP.

## 1. Introduction

### 1.1. Parkinson’s Disease

Parkinson’s disease (PD) is a chronic neurodegenerative disorder that results from the progressive death of dopaminergic neurons located in the substantia nigra and resultant degeneration of dopaminergic pathways in the basal ganglia [1,2,3]. The loss of dopamine alters both inhibitory and excitatory pathways, resulting in the PD cardinal motor signs: bradykinesia (slowness of movement), tremor (trembling in hands, arms, legs, jaw, and face), impaired balance and posture, and muscle rigidity (stiffness of the limbs and trunk) [4,5,6,7]. Other non-motor PD symptoms may include depression and other psychiatric manifestations; difficulty swallowing and speaking; urinary problems or constipation; sleep disruption; and a myriad of other symptoms not overtly seen or displayed [6,7,8,9,10,11].

PD is both chronic and progressive [12]. Symptoms present gradually over several years and the progression varies from person to person, both in rate of development and the extent to which these symptoms manifest. PD dramatically alters an individual’s movement ability and functional stability, affecting not only physical wellness but also quality of life and psychosocial well-being. PD is second to Alzheimer’s disease as the most common age-related chronic, neurological disorder. Currently, over one million people in the United States are living with PD and ~60,000 new diagnoses are made each year [13].

### 1.2. Neurodegenerative Diseases and the Positive Impact of Exercise

Neurodegenerative diseases are characterized by progressive nervous system dysfunction [14,15,16]. The most prevalent neurodegenerative disorders include (listed alphabetically) Alzheimer’s disease (AD), amyotrophic lateral sclerosis, Friedreich’s ataxia, Huntington’s disease, multiple sclerosis, Parkinson’s disease (PD), prion disease, spinocerebellar ataxia, and spinal muscular atrophy. With time, neurodegenerative diseases lead to difficulties either with movement (ataxias) or with mental functioning (dementias). There is increasing evidence for a positive role of exercise in improving both the quality of life and symptoms of many neurodegenerative disorders, especially PD and AD [17,18,19,20,21]. Since the etiology of neurodegenerative disorders is both broad and complex, the expected effect of exercise varies among the disorders. Our focus moving forward is centered on PD and reducing the risk of falls using exercise, primarily, from hitting golf balls and playing golf.

### 1.3. Maintaining Balance in the Presence of Parkinson’s Disease

Posture, balance, and axial mobility are the foundation upon which many day-to-day activities are executed, typically, without conscious thinking. Stopping to bend over and tie your shoes on a walk, or turning to look over your shoulder when driving requires a certain degree of axial movement, flexibility and postural reaction in order to maintain the body’s equilibrium in relation to external forces [22]. Postural instability refers to an impaired ability to maintain posture and balance, compromising the capability to easily and efficiently maintain or change positions [23]. One of the cardinal symptoms of PD, postural instability presents in many patients and worsens as the disease progresses [23,24].

### 1.4. Can the Game of Golf Reduce Falls in Older Adults with Parkinson’s Disease?

**Figure 1:**
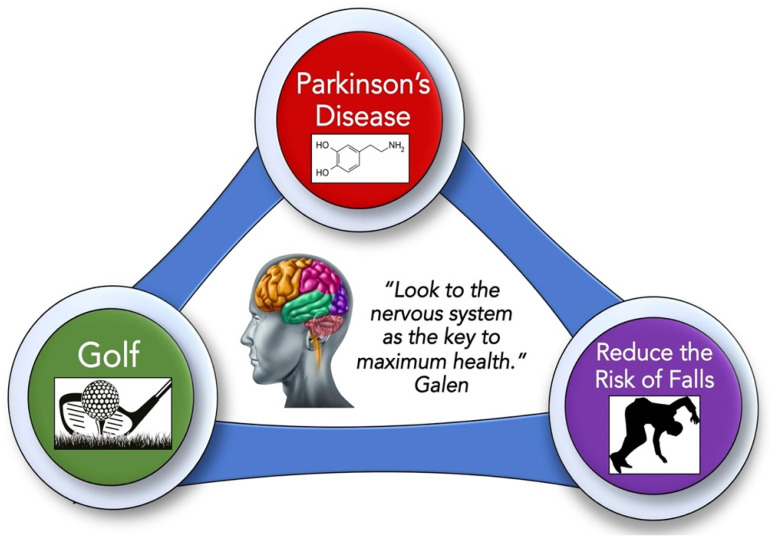
Playing golf may lessen the risk of falls in older adults with Parkinson’s disease (PD). The goal of this narrative review is to highlight the potential role of golf to reduce falls in older adults with PD. The flow of this paper follows the schematic above by describing Parkinson’s disease, a chronic neurodegenerative disease. Over time, PD develops due to the loss of dopamine-producing neurons in the brain (the chemical structure of dopamine is shown above), which greatly diminishes balance, posture and mobility. Older adults with PD have a substantially increased risk for falls compared to age-matched adults without PD. Our literature review found that golf could be an ideal form of exercise for lowering the risk of falls in older adults in general, but especially for those living with PD. The quote is attributed to Aelius Galenus (Anglicized to Galen). He was a prominent Greek physician-philosopher in the ancient Roman Empire who studied many aspects of medicine including anatomy and neurology [29].

Evidence suggests older adults are at increased risk for falls [25,26]. Likewise, there is additional indication that people (person)-with-Parkinson’s disease (PwP) have a substantially higher risk of falls compared to age-matched non-Parkinson’s people [27,28]. In this narrative review, from our summary of the literature, we suggest that older adults with PD who routinely hit golf balls could possibly lower the chance of falling (Figure 1).

## 2. Increased Risk of Falling in Older Adults

### 2.1. Causes of Falls

Falls are one of the leading causes of morbidity and mortality in older adults [25,26]. Importantly, the risk of falling increases with age [26]. The majority of falls typically are caused by a constellation of conditions, including balance and gait deficits, visual impairment, postural hypotension, dementia, neurologic and musculoskeletal disabilities, medications, and environmental hazards (see [30] and references cited therein). In a separate study, Ambrose et al. [31] found that the major risk factors predicting falls were impaired balance and gait, history of previous falls, and polypharmacy. Globally, the most common type of injury reported after a fall were lower extremity fractures including the patella, tibia, fibula, or ankle [32]. However, older adults who have fallen experience increased prevalence of wrist and hip fractures, in addition to potentially serious complications such as head injuries if they are concurrently being treated with oral anticoagulants [33,34,35,36].

Moreland et al. [37] found that lower muscle weakness was a predictor of increased risk of falls in older adults. Balance ability is a key part of our daily life and can be divided into two types: static and dynamic. As we age, controlling balance declines from changes in the vestibular, visual, somatosensory, musculoskeletal, and central nervous systems [38]. In assessing risk for falling in an elderly population, Dunsky et al. [39] recommended both static and dynamic tests used for determination of balance ability.

An algorithm has been developed by the Centers for Disease Control and Prevention [40] to enable health care workers to help older adults with a history of falls: “Multifactorial interventions should include exercise, particularly balance, strength, and gait training; vitamin D supplementation with or without calcium; management of medications, especially psychoactive medications; home environment modification; and management of postural hypotension, vision problems, foot problems, and footwear.”.

### 2.2. Role of Exercise to Lower the Risk of Falls

Lee et al. [41], presented in a prescription-based format, that older adults would gain many health benefits from aerobic exercise, strength or resistance training, flexibility or stretching exercises, and balance training. In a comprehensive review of over 50 trial studies, Sherrington et al. [42] provided guidelines for how exercise can be used to lower the occurrence of falls in older adults. They suggested that exercise should be ongoing and continual for at least 2 h/week, and both the general and high-risk population groups be targeted for exercise programs [42]. Importantly, they recommended that high-risk individuals not be asked to do brisk walking.

Lesinski et al. [43] designed and implemented a balance training program using healthy community adults; furthermore, those enrolled in this program improved static/dynamic steady-state, proactive, and reactive balance. Normal aging is associated with reduced capacity and loss of strength with musculoskeletal functioning. Furthermore, as we age, we generate more sedentary time. Any increase in physical activity is better than being sedentary. Gschwind et al. [44] evaluated a fall prevention program that contained exercises of balance, strength, and power; combined with cognition, psychosocial well-being, and self-efficacy of falls in healthy older adults.

## 3. Parkinson’s Disease and the Increased Risk of Falls

### 3.1. PwP Have an Increased Probability of Falling

PwP are nine times more likely to fall compared to age-matched healthy individuals [45]. PwP have been reported to fall frequently, ~70% report falling yearly, and sadly, 13% report falling weekly [46,47,48]. Due to the average age of PwP (≥65 years old), the same risk factors for falls described in the previous section exists in PD. However, due to many circumstances, PwP must confront a daunting list of issues that predispose them to falls. Shown in Table 1 are some of the compiled risk factors for falls in PwP. These risk factors impact balance, mobility, muscle weakness, and strength, and could affect an individual’s ability to participate in sport. For a more complete review of the various factors that promote falls in older adults with PD, please see [28,45,49] and references cited therein.

### 3.2. Exercise Has Proven Beneficial in Reducing Falls in Parkinson’s Disease

Fall prevention programs using exercise and motor training have been devised to reduce falls in PD [63,64]. Two clinical trials targeting balance resulted in a statistically significant reduction in falls [65,66]. Alternatively, under similar exercise conditions, two other studies did not find a statistically significant reduction in falls [67,68]. Under supervision, Tai Chi exercise focused on flexibility and leg strengthening, resulted in improved anticipatory balance in PwP [69,70]. Three separate studies focused on gait and balance training [71], strategies of fall prevention [72], and balance and strengthening with fall prevention advice [73] showed improvement in fall reduction following therapy. We are making progress in understanding the complex clinical picture of falls in PD [74,75]. Although positive results have been reported, there is clearly a need for additional exercise programs to help reduce falls in PD. We turn our attention to golf. Several components of the game of golf with PD and falls are discussed moving forward.

## 4. Golf as an Exercise to Reduce Falls in Older Adults

### 4.1. Overview of Golf Compared to PD-Specific Exercises

Positive research results have shown the effect of exercise on the alleviation of physical and neurological PD symptoms [76,77,78,79,80,81,82,83,84,85]. This has led to the development of PD-specific exercise programs designed to improve cardinal physical symptoms. Examples of PD-specific exercise programs include PWR! Moves, Rock Steady Boxing and Dance for PD programs, power walking with poles, stationary biking, tai chi, and yoga [86,87,88,89,90,91,92].

The game of golf is not only physical exercise but, as a game of constant strategy, it provides an opportunity for continual cognitive involvement, and it is a sport of camaraderie and social support [93,94,95,96,97,98,99]. However, the unique combination of axial rotation, muscle strengthening, cognitive, and social components obtained through playing golf has yet to be fully explored as an exercise treatment regimen to reduce the risk of falls for older adults with PD.

### 4.2. The Golf Swing

**Figure 2:**
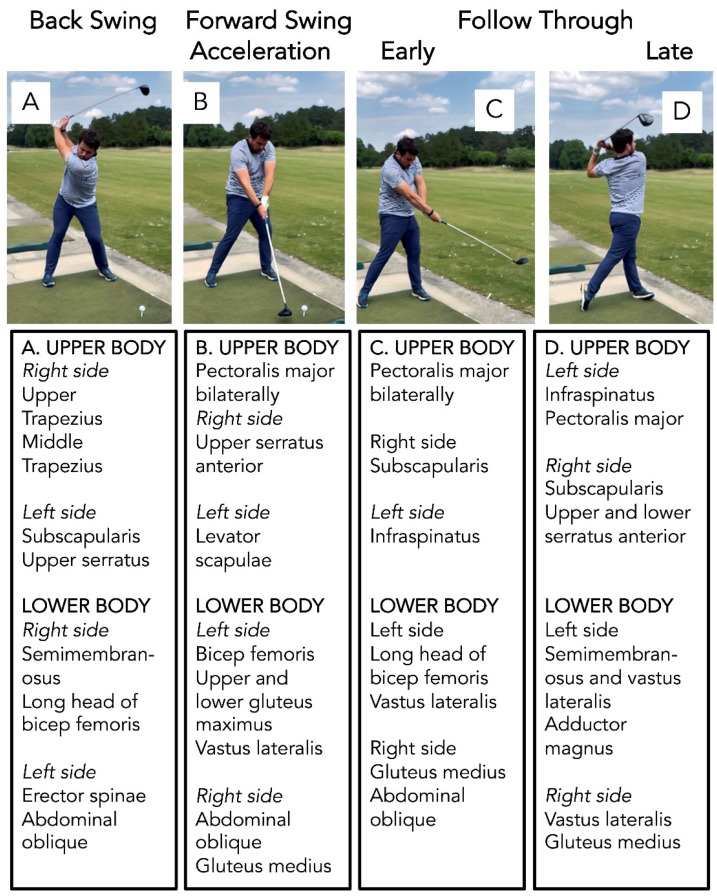
The major muscles used in the golf swing. Shown in (**A**) (far-left panels) are the major muscles used for the back swing, (**B**) (left-middle panels) are the major muscles used for the forward acceleration phase of the down swing, and (**C**) (right-middle panels) and (**D**) (far-right panels) are the major muscles used for the early and late phases of the follow through, respectively. Muscles highlighted above were from the work of McHardy and Pollard [107].

Golf is a sport that offers an all-around low-impact exercise [100,101,102]. The golf swing is a whole-body goal-oriented task that requires an extension, axial mobility, weight shift, coordination, transition of the body (over the base of support), and focus on visual-motor integration to allow the golf club to hit the golf ball [103,104]. The golf swing can be split up into five segments: (i) back swing (ball address to top of back swing); (ii) forward swing [top of swing to club horizontal (early part of down swing)]; (iii) forward swing acceleration [horizontal club to impact (late part of down swing)]; (iv) early follow through (impact to horizontal club); and (v) late follow through (horizontal to completion of swing) [105,106,107]. The complete golf swing not only promotes full range of bodily motion and axial rotation flexibility but activates many muscles in the body [100,101,102,103,104,105,106,107,108,109,110,111]. Shown in Figure 2 are the various major muscles used in the golf swing [107].

**Figure 3:**
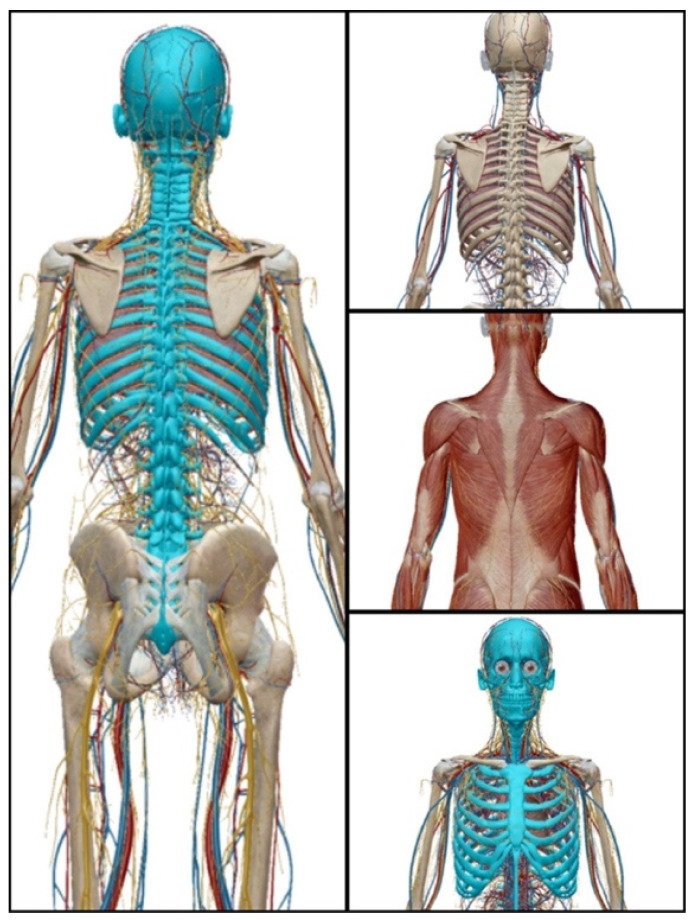
The axial skeleton region (highlighted in blue) along with the surrounding neurovascular and muscle supporting structures. This figure was prepared with permission from Human Anatomy Atlas (Version 2021) [iPhone software. from www.visiblebody.com (accessed on 7 April 2021)].

Essential to the proper execution of the golf swing is the axial skeleton [22,112]. The axial skeleton (highlighted in blue in Figure 3) includes all the bones along the body’s long axis, including the skull, laryngeal skeleton, vertebral column, and thoracic cage. The appendicular skeleton includes all the bones from the upper and lower limbs and the shoulder and pelvic girdle; these bones “append” to the axial skeleton. The axial structures form the supporting platform for the movement of the head and the limbs. Thus, axial mobility can alter our ability to move adjacent joints comprising the shoulder and the pelvic complexes [22,112]. From practicing the golf swing, one could improve spinal rotation to increase axial mobility. Therefore, we speculate that routinely practicing the golf swing could counter inadequate spinal rotation caused by reduced axial mobility, which is one of PD’s consequences.

### 4.3. Playing Golf Promotes Wellness and Reduces Falls in Older Adults

Tsang and Hui-Chen [113] found that older golfers had better static and dynamic balance control compared to the control group of older healthy non-golfers. Next, Gao et al. [114] suggested that the combined precision and repetitive nature of the golf swing and walking the golf course favored older golfers to have better balance and more confidence when compared to the control group of older healthy non-golfers. Comparing experienced older golfers to older Tai Chi practitioners, Tsang and Hui-Chen [115] reported that both groups had better joint proprioception and balance control during weight shifting compared to age-matched controls. Furthermore, both older golfers and Tai Chi test subjects were comparable in balance control to healthy college-aged control subjects [115].

Du Bois et al. [116] gave golf lessons to two older adults who were non-golfers and they measured significant increase in strength and power along with improved balance and posture. Martinez-Bustelo et al. [117] measured quadriceps symmetry in active, sedentary, and institutionalized lifestyle in females over 80 years of age. The active group were golfers and they had increased muscular function compared to the other groups [117]. Du Bois et al. [118] used a 12-week comprehensive golf training program to study the physical abilities, dynamic balance, and hip muscle performance of older military veterans. They found that the older military veterans showed improved physical performance and dynamic balance from playing golf [118].

In a direct comparison of golf to Tai Chi for patients with moderate PD, Johnson et al. [119] reported golf was associated with greater improvements in balance and mobility than Tai Chi. Murray et al. [98] reviewed golf and health. Besides finding improved physical health and mental well-being in golfers, they also suggested that playing golf contributes to muscle strengthening, improved balance and fall prevention [98]. Collectively, these studies reinforce the hypothesis that golf [(a) repetitively hitting golf balls; (b) playing a round of golf; and (c) walking while you play golf] strengthens the body, improves posture, coordination and axial rotation, and enhances balance. These studies imply that older adults who routinely practice/play golf should be less likely to fall.

## 5. Golf Could Improve Functional Ability in Older Adults with Parkinson’s Disease

### 5.1. Comparing Regular Physical Activity to Playing Golf

Individuals with PD who exercise consistently have been shown to preserve function, slow disease progression [120], demonstrate positive effects on mobility, and improve health-related quality of life [121]. Regular physical activity has also been shown to have a positive correlation with improved functional capacity, posture, and balance with reduced risk for falls in PwP [122]. As previously described, golf offers the potential to improve axial rigidity, bradykinesia and postural instability due to qualities inherent to the swing and game of golf itself. Furthermore, components of strategy, social engagement, and focus on task offer potential to address non-motor symptoms and improve beneficial carryover for PwP. We now highlight specific aspects of the golf swing for improving postural instability in PD, and we describe some golf-specific activities for PwP.

### 5.2. Postural Instability: Anticipatory Movements and Bradykinesia

Postural instability in PD is a leading cause of falls throughout the disease progression, and in advanced stages, can lead to a loss of independence [123,124]. This is of particular concern because postural instability does not always respond positively to medications used to manage symptoms of PD, highlighting the importance of identifying salient strategies to heighten postural reaction [123,125]. King and Horak [126] examined lateral stepping strategies in individuals with PD (both on and off medication) and found that individuals with PD had a reduced ability to recruit anticipatory postural adjustments (APAs) characterizing their steps as smaller, and slower with increased prevalence for falls as compared to age norms. Medication did not significantly impact falls or stepping strategy. Furthermore, Lin et al. [127] showed that bradykinetic APAs and varied force production may contribute to walking hesitation and potentially increase falls in PwP. A study by Dijkstra et al. [128] showed that reduced amplitude during weight shift was associated with freezing of gait, a pronounced sign of postural instability in advanced stages. The golf swing has a forceful and quick weight shift that is applied laterally during the early down swing and followed by a sustained weight shift onto the opposite limb during the follow through [108]. We reason that this action has potential to address bradykinesia in PD with anticipated movement in a lateral direction, and potentially improve postural instability with repetition.

### 5.3. Postural Instability: Axial Rigidity and Coordination

A forward flexed posture, truncal rigidity, and limited trunk mobility are common tenets in a PwP and further contribute to postural instability [123]. Coactivation of agonist and antagonist muscles on both sides of the body contributes to increased stiffness in the lower legs, hips and trunk leading to ineffective balance recovery [129]. Carpenter et al. [130] used EMG electrodes to assess muscle activation with multidirectional perturbations. They concluded that individuals with PD demonstrated reactive strategies responding to disequilibrium [130]; however, these strategies were inefficient at preventing loss of balance due to a stiffening response in the ankles, trunk, and hips. Components of the golf swing require extensive thoracolumbar rotation while controlling the momentum and weight of the golf club to be performed successfully. We propose this coupling of the golf club with rotational movement offers potential to improve axial mobility and trunk rotation in PwP who play golf regularly. Use of the golf club simultaneously offers a force for overpressure to achieve increased stretch and potential for an added perturbation as the golfer controls the movement of the golf club.

Activation of spinal extensors during the golf downswing and follow through complement the need to strengthen antigravity musculature to address stooped posture. Furthermore, neuromuscular coordination between spinal extensors and oblique musculature as the golfer transitions from backswing to down swing offer opportunities for the PwP to practice selective trunk engagement with attention to muscle coordination, potentially limiting the prevalence of co-contraction of trunk musculature during the golf swing. This is of importance because multiple studies have shown that PwP have difficulty with multi-segmental coordination, particularly, coordinating the arm, hand, and trunk movements with reaching tasks [131,132]. Individuals with PD may be challenged switching from one movement to a different movement. Thus, the transitional movement between set up, back swing and down swing of the golf club may facilitate opportunities for PwP to practice weight shift and transitional movements.

### 5.4. Motivation and External Engagement

Several studies have demonstrated the benefit of PwP exercising with increased intensity in addition to use of cues to augment motor learning [133,134,135]. This can be challenging to achieve without the guidance of a physical therapist or exercise instructor specializing in PD. Although golf does not replace these components of an individual’s healthcare team, golf offers opportunity to utilize external feedback of how far or fast the ball moves to improve the individual’s ability to assess their own performance and adjust accordingly. Motivation to hit the ball far on a long drive may facilitate increased weight shift, amplitude, and power beyond what the individual may have completed without the external cue of a ball.

The importance of movement and exercise are becoming increasingly understood as an important strategy for minimizing disease progression in people with PD. However, creating a habit of daily exercise can be a challenge for individuals with chronic disease who may be impacted by non-motor symptoms of reduced motivation or depression. A report by Chong et al. [136] found that even in individuals who were not clinically depressed, individuals with PD were less motivated than controls if rewards were low. Golf offers the opportunity to increase motivation with the use of a goal, social engagement, and connection with the outdoors. Interestingly, Johnson et al. [119] found that participants with PD were more likely to continue with golf (86%) as compared to Tai Chi (33%) at the end of the study. Furthermore, a full game of golf requires strategy and planning, offering the potential to improve cognitive skills including executive function, which can be impacted in PD.

### 5.5. Hitting Golf Balls and Playing Golf

Gary Smith has PD and is using golf as part of his exercise therapy (from Golf Channel, “Comeback: Smith battles Parkinson’s disease with golf” https://www.golfchannel.com/video/comeback-smith-battles-parkinsons-disease-golf/ (accessed on 16 April 2021)). He started by hitting 100 golf balls/day at a TopGolf facility and over the past several years, his medical therapy has not advanced. Typically, increases in medication dosages imply disease progression.

Four strategies for hitting golf balls are described below (Table 2). While reports of golf reducing falls in older adults are both positive and encouraging, they comprise a handful of publications (with only one study pertaining to PD) [113,114,115,116,117,118,119]. Thus, based on these previous studies, current understanding of PD, and the biomechanics of a golf swing, we speculate and offer some expected goals/outcomes for PwP while using the golf plans detailed in Table 2.

Plan #1 involves practicing the golf swing at home. As described above (see Figure 2 and Figure 3), the proper golf swing utilizes many muscles in the upper and lower body. Furthermore, the mechanics of the golf swing offer potential to improve anticipatory postural adjustments and weight shift ability, in addition to coordination, strength, and mobility of trunk musculature. These components may provide a helpful mechanism to reduce the risk for falls (See Section 5.1 and Section 5.2 above for discussion at length of these concepts and related research). With repetition, balance and coordination should improve. Current literature and anecdotal accounts are limited and describe a varied frequency from 2 x/week [119] to 5–7 x/week [see Gary Smith noted above]. Therefore, we propose an exercise dose and frequency of hitting 100 golf balls 3–5 x/week (a typical hour at the driving range involves hitting 50–100 golf balls), although more research is needed to determine optimal dose and frequency.

Plan #2 involves hitting golf balls at a golf driving range. In addition to the benefits discussed in the previous plan, the golfer will now have additional feedback on how far the golf ball travels. As previously discussed in Section 5.4 above, use of external cues may enhance motor learning performance in a PwP [133,134,135] and motivate the golfer to move beyond self-selected effort.

Plan #3 and plan #4 have PwP playing a round of golf on a real surface and virtually, respectively. These golf plans both add components of strategy, cognitive engagement, and a social setting for PwP, allowing the sport to have a positive impact that is multidimensional with potential to address non-motor symptoms in addition to the benefits previously discussed. Playing on a real golf course offers the opportunity to walk on uneven surfaces which may further optimize dynamic balance [114]. With time and continued practice, the golfer with PD should realize improved posture, coordination, balance, and enhanced axial spine rotation (as discussed in Section 5.3 above).

The golf plans described here offer a framework for another exercise strategy for PwP to gain strength, improve balance, coordination, posture, and axial rotation, thereby, potentially reducing their risk of falls. While golf may be considered an expensive sport, plan #1 and plan #2 provide relatively inexpensive options to improve access and allow PwP to benefit from the full-body exercise workout that swinging a golf club provides. Other sports, such as boxing, tai chi, dance, yoga, and pickleball, were also once considered relatively expensive sports that were not easily accessible or available to adapt for PD. However, all of these exercises have inspired community programs and become widely used by PwP to improve quality of life and offer relief from their motor symptoms. Our goal is to “plant-a-seed” for golf, inspire further research, give rise to community programs, and hopefully, watch the game of golf grow in use by PwP as an engaging past time that may prevent falls associated with complications from PD.

## 6. Conclusions

PD is a chronic neurodegenerative disorder featuring the development and progression of both motor and non-motor defects. This narrative review presents golf as a physical activity to lower the risk of falls in community ambulating older adults with PD. Our literature review has shown that many types of exercise can lessen the risk of falls in older adults. The biomechanics of the golf swing supports the concept of strengthening the upper and lower body, increasing the process of balance, enhancing axial mobility, and improving coordination and posture, all of which should help anyone with PD. Golf also has the potential to improve the quality of life for PwP. Although further study is needed, playing golf regularly may be beneficial in reducing the risk of falls in older adults with PD.

## Figures and Tables

**Table 1:** Risk factors for falls in Parkinson’s disease.

Risk Factor	Reference(s) Cited
**Exercise/Sports-Related:**	
Balance-mobility	[47,48,50,51,52]
Decreased arm swing	[48]
Gait disturbances	[28,47,50]
Inability to get up from a sitting position	[28,53]
Orthostasis	[28,47]
PD-specific clinical symptoms:	
Bradykinesia	[53]
Dyskinesia	[51]
Freezing	[51,52,54,55,56,57]
Rigidity	[50,58]
**General Neurological/PD-Related:**	
Advanced age	[53,59]
Cognition	[57,60,61]
Depression and anxiety	[48,50,62]
Disease severity	[51,52,56]

**Table 2:** Golf exercise routines for Parkinson’s disease.

**Be safe, be careful, and realize that most PwP typically have gait and balance issues**
To ensure safety, the benefit of a new exercise program will only work if you have (i) talked with your neurologist; (ii) worked out a plan with your physical therapist or personal trainer that includes stretching exercises for pre- and post-golf and discussion on the optimal dose and frequency as you prepare for a new exercise; (iii) if you know how to play golf and you are starting anew, welcome back; and (iv) if you are a beginner golfer, welcome, and have fun.
**Plan #1: Practice the golf swing at home**
This plan requires only a couple of golf clubs, a golf mat to swing on and likely plastic golf balls (and possibly a net to capture balls). If weather permits, practice outside. The goal is to swing the golf club 100 times/day for 3–5 days per week.
**Plan #2: Hitting golf balls at a golf driving range**
The next level is going to a golf driving range, where you are hitting real golf balls and likely have a few more golf clubs. Similar goal, try to hit 100 golf balls/day for 3–5 days per week.
**Plan #3: Play a round of golf at a local golf course/club**
The third level is to play a round of golf, playing either 9 holes or 18 holes at a local golf course. If you are able to walk, you enhance the exercise routine. Try to play once (or twice) per week. Golf has many rules, yet it is a very honorable and social sport. You can enjoy it playing alone or with friends. Two downsides to golf include the time it takes to play 9 or 18 holes of golf, and it is not without significant equipment and clothes expenditures. However, plans #1 and #2 described above are much more reasonable in terms of time and cost.
**Plan #4: Virtual Golf**
“Park Place Golf Club” is designed specifically for Parkinson’s disease. This is a virtual 18-hole golf and fitness training program that provides instructional golf videos paired with functional fitness workouts where you can track your progress. Park Place Virtual Golf was created by Taunya Foerster [from https://parkplacegolfclub.com (accessed on 16 April 2021)].
**Learning how to play golf**
“Golf for Beginners: So You Want To Play Golf” [from https://www.golfdigest.com/gallery/golf-beginners-tips/amp (accessed on 16 April 2021)]; “Beginner Basics” [from https://schools.golfdigest.com/series/will-robins-beginner-basics-rsuzob7 (accessed on 16 April 2021)]; “The best way to learn golf now” [from https://www.golfdigest. com/story/how-to-play-golf-now/amp (accessed on 16 April 2021)]; “A Beginner’s Golf Guide: What every new golfer should know when picking up the game” [from https://www.golfdigest.com/story/the-complete-beginner-s-guide-to-golf (accessed on 16 April 2021)]; “Golf 101: Dos and don’ts for beginners” [from https://golf.com/instruction/golf-101-dos-and-donts-for-beginners/ (accessed on 16 April 2021)]; “How to Play Golf | The Beginner’s Guide” [from https://golfworkoutprogram.com/how-to-play-golf/ (accessed on 16 April 2021)].

## Data Availability

Not Applicable.
